# Emotional disorders in the structure of psychoorganic pathology in tumors of the diencephalon

**DOI:** 10.1192/j.eurpsy.2024.752

**Published:** 2024-08-27

**Authors:** Y. Sidneva, L. Astafyeva, O. Zaitsev, P. Kalinin, M. Kutin, A. Shkarubo, D. Fomichev, I. Voronina, D. Andreev, O. Sharipov, I. Chernov, I. Klochkova, I. Badmaeva, A. Donskoy

**Affiliations:** ^1^Neuropsychiatric research, N.N.Burdenko National Medical Research Center of Neurosurgery; ^2^Rehabilitation, Clinical and Research Institute of Emergency Pediatric Surgery and Trauma; ^3^Neuroendocrinology; ^4^Neurosurgery, N.N.Burdenko National Medical Research Center of Neurosurgery, Moscow, Russian Federation

## Abstract

**Introduction:**

The tumors of the diencephalon region (thalamic-hypothalamic-pituitary system) include a large group: pituitary adenomas, craniopharyngiomas, gliomas, and others. Tumors differ in the histological structure, and manifestations of the clinical symptoms; by hormonal data; by approaches and methods in treatment.

Psychic symptoms are revealed in disease in addition to cerebral, neuroendocrine symptoms, neurological disorders. Psychoorganic syndrome is represented by emotional, motivational, personal, cognitive impairments, inversion of the sleep-wake cycle, seizures.

Disorders of mental activity are detected in all tumors of this localization in varying degrees, according to the different authors from 20 to 100%; affective pathology varies from 2 to 80% by the literature.

**Objectives:**

To study the emotional disorders in the structure of psychoorganic pathology in tumors of diencephalon region

**Methods:**

290 patients (18-78 years old, mean age 38+2): pituitary adenomas (PA), as the most common – 170 (58,6%), craniopharyngiomas (CG), as with the most varied manifestation of mental symptoms – 120 (41,4%). Methods: psychopathological, data from endocrinological, neurological, neuroimaging methods.

**Results:**

Emotional disorders were detected in patients from 30 to 68% of cases, depending on the histology of the tumours: PA with excessive secretion of growth hormone - emotional disorders are in 60%; PA with excessive secretion of adrenocorticotropic hormone - in 50%; PA with excessive secretion of prolactin - in 30%; with excessive secretion of thyroid-stimulating hormone - in 40%; non-functioning PA - in 16%; CG - in 68%.

Emotional disorders were more often represented by changeable mood, depression, apathy, sleep disturbance, and visceral symptoms. Symptoms differed depending on the histology of the tumor (type and level of hormones), the volume of the lesion and direction of growth, and concomitant hypertensive-hydrocephalic symptoms. Emotional disturbances often include memory impairment, personality and behavior changes.

**Conclusions:**

Emotional disorders are detected in patients in 30-68% of cases in the structure of psychoorganic pathology with damage to the diencephalon region (in particular, with pituitary adenomas and craniopharyngiomas); are determined by the topography of the tumor and histology with the involvement of the corresponding structures and nuclei in the pathological process.

**Disclosure of Interest:**

None Declared

